# FOXP3+ T Cells—An Emerging Evidence in Periodontitis Therapeutics

**DOI:** 10.1002/cre2.70263

**Published:** 2025-12-15

**Authors:** Revan Birke Koca Ünsal, Akira Hasuike, Tamer Badawy, Farah Asa'ad, Bruno Špiljak, Monal Yuwanati, Jelena Roganović, Yinli Liu, Carel Brigi, Danilo Milanes Zambrano, Akhilanand Chaurasia

**Affiliations:** ^1^ Schulich School of Medicine & Dentistry Western University London Canada; ^2^ Department of Periodontology Nihon University School of Dentistry Tokyo Japan; ^3^ Department of Oral Biology, Faculty of Dentistry Cairo University Cairo Egypt; ^4^ Department of Oral Biology, Faculty of Dentistry Galala University New Galala City Egypt; ^5^ Department of Oral Biochemistry, Institute for Odontology The Sahlgrenska Academy at University of Gothenburg Gothenburg Sweden; ^6^ School of Dental Medicine University of Zagreb Zagreb Croatia; ^7^ Department of Oral and Maxillofacial Pathology, Saveetha Dental College and Hospitals, Saveetha Institute of Medical and Technical Sciences Saveetha University Chennai India; ^8^ Faculty of Dental Medicine University of Belgrade Belgrade Serbia; ^9^ Department of Orthodontics, Academic Centre for Dentistry Amsterdam (ACTA) University of Amsterdam and Vrije Universiteit Amsterdam Amsterdam The Netherlands; ^10^ Department of Oral Diagnosis, Research Institute for Medical and Health Sciences University of Sharjah Sharjah UAE; ^11^ Department Pediatric Dentistry and Orthodontics, College of Medicine and Health Sciences University of Rwanda Kigali Rwanda; ^12^ Department of Oral Medicine and Radiology King George's Medical University Lucknow India

**Keywords:** FOXP3+ regulatory T cells, immunomodulation, periodontal diseases, periodontitis, Tregs

## Abstract

**Objective:**

To review interaction of FOXP3+ regulatory T cells with Th17 cells in determining the progression of periodontitis.

**Material and Methodology:**

Literature review pertaining to FOXP3+ regulatory T cells, Th17 cells, and periodontitis was analyzed. Descriptive summary is presented.

**Results:**

FOXP3+ regulatory T cells (Tregs) play an essential role in maintaining immune homeostasis and modulating inflammatory responses. The balance between Tregs and pro‐inflammatory Th17 cells is crucial in determining the progression of periodontitis, a chronic immune‐mediated inflammatory disease. While Tregs are responsible for suppressing excessive immune activation and preventing tissue destruction, an imbalance favoring Th17 cells leads to increased osteoclastic activity and alveolar bone loss through IL‐17 and RANKL signaling. The inflammatory microenvironment in periodontitis compromises FOXP3+ Treg stability and function, thereby allowing unregulated immune responses that exacerbate periodontal tissue breakdown. Recent studies suggest that strategies aimed at enhancing Treg‐mediated immune regulation, such as IL‐2 supplementation, all‐trans retinoic acid (ATRA), IL‐33 administration, and CCL22‐mediated recruitment, could mitigate periodontal inflammation and preserve alveolar bone integrity. Furthermore, systemic conditions like diabetes and obesity play a significant role in disrupting Treg function by promoting a pro‐inflammatory environment, impairing immune regulation, and exacerbating immune dysregulation. This dysfunction weakens the protective role of Tregs, leading to an intensified inflammatory response that accelerates periodontal tissue destruction and alveolar bone loss.

**Conclusion:**

Understanding the mechanisms governing FOXP3+ Treg stability and their interaction with pathogenic Th17 responses is essential for developing targeted immunomodulatory therapies. Future research should focus not only on selectively expanding Tregs but also on translational strategies such as adoptive Treg transfer and IL‐17 inhibition, while carefully balancing efficacy and the risk of systemic immunosuppression.

## 1 Introduction

### 1.1 Periodontitis as a Chronic Inflammatory Disease

Periodontitis is a chronic inflammatory disease that progressively destroys tooth‐supporting structures, including the periodontal ligament, cementum, and alveolar bone (Könönen et al. 2019; Sedghi et al. 2021). It results from a complex interaction between a dysbiotic microbial biofilm and the host immune system in genetically and environmentally susceptible individuals (Hajishengallis 2014; Loos and Van Dyke 2020). The microbial biofilm, primarily composed of Gram‐negative anaerobes such as *Porphyromonas gingivalis, Treponema denticola*, and *Tannerella forsythia*, triggers an exaggerated immune response (Hajishengallis 2014; Socransky and Haffajee 1991; Abdulkareem et al. 2023).

Although this immune response is initially aimed at controlling the microbial challenge, it paradoxically contributes to tissue destruction and disease progression by driving persistent inflammation and bone resorption (Martínez‐García and Hernández‐Lemus 2021). Key mechanisms include the overproduction of pro‐inflammatory cytokines such as TNF‐α, IL‐1β, and IL‐6, along with osteoclast‐mediated bone resorption (Terkawi et al. 2022). Additionally, matrix metalloproteinases (MMPs), particularly MMP‐8 and MMP‐9, degrade collagen in the periodontal ligament and extracellular matrix, while reduced levels of tissue inhibitors of metalloproteinases exacerbate tissue destruction (Kornman 2008).

Unlike acute inflammation, which resolves after pathogen elimination, chronic inflammation in periodontitis persists due to an imbalance in pro‐inflammatory and anti‐inflammatory pathways, leading to continuous tissue damage (Martínez‐García and Hernández‐Lemus 2021; Cekici et al. 2014). Neutrophils and macrophages further exacerbate tissue breakdown by releasing reactive oxygen species (ROS) and nitrogen species, impairing tissue healing (Patil et al. 2024).

The oral microbiome plays a critical role in immune modulation during periodontitis (Abdulkareem et al. 2023). Dysbiosis fosters an environment that allows keystone pathogens like *P. gingivalis* to evade immune responses through complement subversion and Toll‐like receptor (TLR) signaling pathways (Hajishengallis and Diaz 2020; Lamont et al. 2018).

Targeted therapies, including host‐modulation agents (e.g., sub‐antimicrobial dose doxycycline), probiotics, and regenerative biomaterials, aim to modulate inflammation and restore microbial balance (Golub and Lee 2020). However, growing evidence suggests that the key to controlling periodontal destruction lies in the balance between pro‐inflammatory Th17 cells and immunosuppressive FOXP3+ regulatory T cells (Tregs). An imbalance skewed toward Th17 activity contributes to tissue destruction, whereas FOXP3+ Tregs suppress excessive immune activation and promote tissue repair. Systemic conditions such as diabetes and obesity further disrupt this equilibrium, exacerbating disease progression (Loos and Van Dyke 2020; Abdulkareem et al. 2023; Martínez‐García and Hernández‐Lemus 2021; Cekici et al. 2014; Golub and Lee 2020).

As chronic inflammation persists due to immune imbalance, attention has therefore turned to regulatory mechanisms that maintain immunological tolerance, particularly FOXP3+ Tregs. These cells may play a pivotal role in mitigating periodontal tissue damage, offering a novel avenue for host‐modulating therapies.

### 1.2 Treg/Th17 Balance in Periodontal Diseases

The balance between regulatory T cells (Tregs) and T‐helper 17 (Th17) cells plays a crucial role in periodontal tissue homeostasis. Tregs, characterized by the transcription factor FOXP3, suppress excessive inflammation and promote tissue repair through anti‐inflammatory cytokines IL‐10 and TGF‐β (Josefowicz et al. 2012; Gao et al. 2017). Additionally, Tregs regulate immune responses via CTLA‐4, which downregulates dendritic cell (DC) activation (Alvarez et al. 2018).

In contrast, Th17 cells, driven by RORγt, promote inflammation and bone resorption. They produce pro‐inflammatory cytokines such as IL‐17, IL‐22, and GM‐CSF, leading to neutrophil recruitment and osteoclast activation, which contribute to periodontal tissue destruction (Tesmer et al. 2008; Cardoso et al. 2009).

An imbalance favoring Th17 cells over Tregs correlates with severe periodontal disease. Elevated IL‐17 levels in gingival crevicular fluid and tissues enhance osteoclastogenesis and tissue destruction, while reduced Treg function fails to counteract these effects (Gao et al. 2017). Factors such as microbial antigens from *P. gingivalis*, genetic predispositions, and systemic conditions like diabetes influence this imbalance (Martínez‐García and Hernández‐Lemus 2021).

Systemic conditions like diabetes exacerbate this imbalance by promoting a pro‐inflammatory environment that impairs Treg function and enhances Th17‐mediated inflammation (Zhao et al. 2023; Huang et al. 2021). Obesity, linked to chronic low‐grade inflammation, also favors Th17 differentiation (Iwashita et al. 2021). This imbalance can be driven by factors such as microbial antigens from *P. gingivalis*, genetic predispositions, and systemic conditions like diabetes (Martínez‐García and Hernández‐Lemus 2021). For instance, diabetes exacerbates this imbalance by promoting a pro‐inflammatory environment that impairs Treg function and enhances Th17‐mediated inflammation (Zhao et al. 2023; Huang et al. 2021). Similarly, obesity, linked to chronic low‐grade inflammation, favors Th17 differentiation (Iwashita et al. 2021).

Targeting the Treg/Th17 axis has shown promise in periodontal therapy. IL‐17 inhibitors effectively reduce tissue destruction and promote bone regeneration (Huang et al. 2021). Emerging therapies include adoptive transfer of expanded Tregs and microbiome‐modulating probiotics to restore immune balance (Amato et al. 2022; Zhang et al. 2022). Future research may offer novel therapeutic targets for managing periodontal disease.

In the following sections, we further elaborate on Th1/Th17 and Th2/Treg homeostasis, how this balance becomes disrupted in periodontitis, and how emerging therapeutic strategies aim to restore it.

### 1.3 Th1/Th17 and Th2/Treg Homeostasis in Periodontitis

Antigen‐presenting cells (APCs) interact with naïve CD4+ T cells, promoting their differentiation into subsets, including Th1, Th2, Th17, and Treg cells (Campbell et al. 2016). This differentiation is driven by cytokine signals. IL‐12 exposure promotes Th1 differentiation, leading to IFN‐γ production, which enhances neutrophil and macrophage activity (Zhu and Zhu 2020). Th2 differentiation is primarily driven by IL‐4 signaling, which activates the transcription factor GATA‐3 and promotes Th2 lineage commitment (Zhu and Zhu 2020; Meningher et al. 2020). In periodontitis, Th1 responses exhibit context‐dependent functions; while IFN‐γ contributes to microbial clearance and protective immunity, sustained Th1 activation can also promote osteoclastogenesis and tissue destruction (Cavalla et al. 2021; Hajishengallis and Korostoff 2017).

Th17 differentiation is regulated by IL‐6, TGF‐β, and IL‐23. In the presence of IL‐6 and TGF‐β1, Th17 cells co‐produce IL‐17 and IL‐10, exhibiting non‐pathogenic behavior (Korn et al. 2009). The expression of granzymes in Th17 cells has been documented predominantly in pathogenic Th17 subsets and appears to be context‐dependent, for example in autoimmune settings (Lee et al. 2012; Bunte and Beikler 2019). Th17 cells play a dual role in periodontitis, mediating both bacterial defense and bone loss (Tsukasaki et al. 2018).

### 1.4 Differentiation of Tregs

Regulatory T cells (Tregs) maintain immune balance and prevent excessive tissue damage. They achieve this by secreting anti‐inflammatory cytokines (IL‐10, TGF‐β) and suppressing effector T cell activation (Singer et al. 2014). Tregs are classified based on their origin: thymus‐derived Tregs (nTregs) target self‐antigens and prevent autoimmunity, while peripherally induced Tregs (pTregs) develop in response to exogenous antigens, preventing local inflammation (Shevyrev and Tereshchenko 2020).

**Figure 1:**
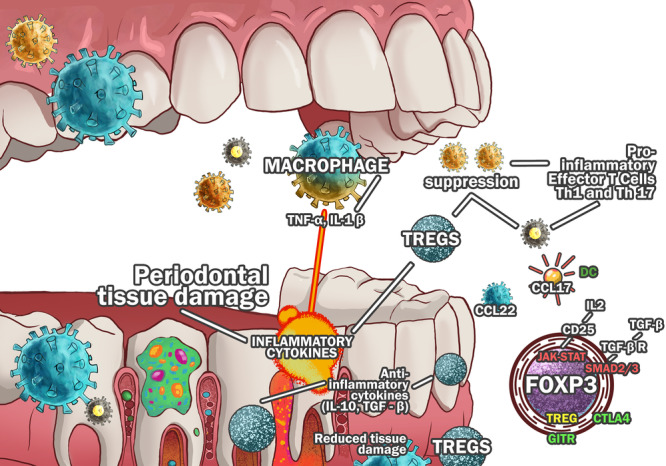
The interplay between macrophages, dendritic cells (DCs), and regulatory T cells (Tregs) plays a key role in controlling periodontal inflammation and tissue destruction. In periodontitis, pro‐inflammatory macrophages release cytokines like TNF‐α and IL‐1β, contributing to tissue damage, while DCs produce chemokines such as CCL17 to recruit Tregs. Tregs help suppress excessive inflammation by inhibiting Th1 and Th17 cells, which drive pro‐inflammatory responses. These cells, whether thymus‐derived or peripherally induced, express CD25, FoxP3, and may also express CTLA‐4 and GITR. Their anti‐inflammatory function relies on cytokines like IL‐10 and TGF‐β, which help regulate immune responses and protect periodontal tissues.

Both nTregs and pTregs express CD25, FoxP3, and lack CD127, but pTregs can lose FoxP3 under inflammatory conditions, differentiating into pro‐inflammatory Th17 cells (Thornton et al. 2010). Tr1 cells and Th3 cells also exhibit regulatory activity but lack FoxP3, producing IL‐10 and TGF‐β instead (Figure 1) (Gagliani et al. 2013). CD8+ Tregs, capable of direct cytotoxic suppression, help regulate immune tolerance and osteoclastogenesis (Han et al. 2018).

Memory Tregs can acquire Th1, Th2, or Th17‐like phenotypes depending on environmental cues. Under inflammation, they may lose suppressive function and promote inflammation instead (Abdeladhim et al. 2022). Tissue migration and adaptation allow Tregs to regulate site‐specific immune responses (Miragaia et al. 2019).

## 2 Tregs in Periodontitis

Tregs maintain immune tolerance and suppress excessive inflammation in periodontitis by inhibiting pro‐inflammatory Th1 and Th17 responses (Huang et al. 2021). However, studies show that Treg function may be impaired in periodontitis, leading to excessive bone loss. Inhibition of Tregs via anti‐GITR results in increased alveolar bone resorption and inflammatory cytokine expression (Garlet et al. 2010; Zhang et al. 2021).

Experimental periodontitis models demonstrate enhanced Th17 and Treg‐related markers, but chronic inflammation can drive Tregs toward a Th17‐like phenotype, exacerbating disease progression (Komatsu et al. 2014). Mechanistically, this plasticity is characterized by downregulation of FoxP3, upregulation of RORγt and acquisition of pro‐inflammatory cytokine secretion (e.g., IL‐17 and IFN‐γ), thus losing suppressive function and contributing to inflammation. These changes are driven by cytokines, such as IL‐6 and IL‐23, and further influenced by genetic predispositions (e.g., HLA‐B27) and environmental factors such as hypoxia (Ortega‐Mejia et al. 2025). Importantly, context‐dependent differences have been observed: in peripheral blood, Th17‐like Tregs may retain suppressive abilities, whereas in inflamed tissues, such as rheumatoid arthritis synovium, they lose regulatory function and can become pathogenic (Wang et al. 2015). Notably, IL‐35 administration has been shown to restore Th17/Treg balance and prevent bone loss by upregulating osteoprotegerin (OPG) (Schmidlin et al. 2021).

Treg migration to periodontal tissues depends on chemokines such as CCL17 and CCL22, which recruit CCR4‐expressing Tregs (Nakajima et al. 2005). Studies indicate an increased presence of Tregs in periodontal lesions, potentially as a compensatory response to inflammation (Tsukasaki et al. 2018). However, FoxP3+ Tregs are diminished in bone resorption sites, highlighting the imbalance between protective and destructive immune responses in periodontitis (Ernst et al. 2007).

A disrupted Th17/Treg ratio accelerates alveolar bone loss, as Th17 cells promote osteoclastogenesis via RANKL, whereas Tregs inhibit it (Ramadan et al. 2020). Additionally, exosomes from periodontal ligament stem cells in periodontitis models enhance Th17 activity while suppressing Tregs, further contributing to bone resorption (Figure 1) (Cai et al. 2023). Recent evidence indicates that periodontal ligament–derived exosomes contain pro‐inflammatory miRNAs such as miR‐155 and miR‐21, as well as cytokines and other bioactive molecules that promote Th17 polarization while suppressing FOXP3 expression in Tregs. These exosomal cargos can reprogram local immune cells by suppressing FOXP3 stability in Tregs and enhancing RORγt‐driven Th17 differentiation, thereby amplifying the inflammatory microenvironment that accelerates periodontal bone resorption (Cai et al. 2023).

### 2.1 Immunomodulatory Role of Tregs in Periodontitis

**Figure 2:**
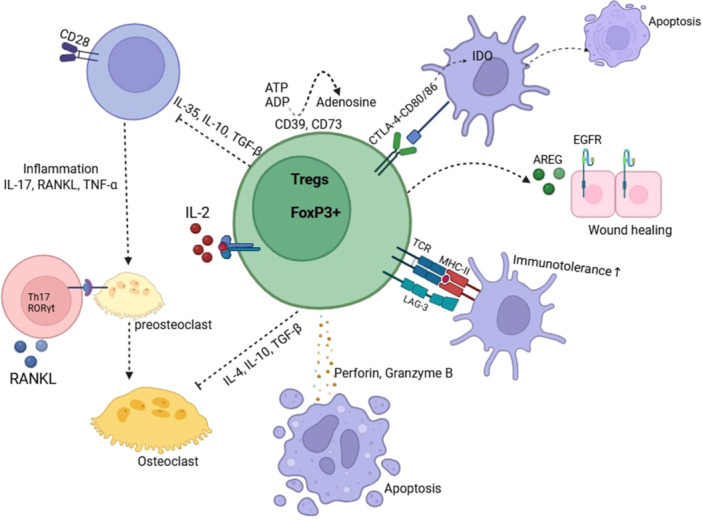
Treg cells have multiple immunomodulatory mechanisms. Tregs inhibit antigen‐presenting cells (APCs) via the inhibitory receptor CTLA‐4, which binds to the costimulatory molecules CD80 and CD86, activating IDO and causing apoptosis. They promote tissue repair through AREG, which acts on EGFR and promotes healing. LAG‐3/CD223 on the Treg surface binds more strongly to major histocompatibility complex (MHC) class II molecules, promoting immunotolerance. They release perforin and granzymes to induce apoptosis in target cells and suppress osteoclastic activity by expressing anti‐inflammatory cytokines IL‐4, IL‐10, and TGF‐β. Tregs also suppress the pro‐inflammatory functions of Th17. CTLA‐4 competes with CD28 present on the surface of effector T cells (Teff) and inhibits costimulatory signals during antigenic presentation. Furthermore, Tregs transform ATP and ADP into adenosine through surface ectoenzymes (CD39 and CD73) to reduce pro‐inflammatory conditions.

CD4+ regulatory T cells (Tregs) play a crucial role in maintaining periodontal homeostasis by mitigating inflammation, preserving gingival integrity, and preventing alveolar bone loss. Their immunosuppressive function primarily involves the inhibition of naive T cell activation and proliferation through both cell‐contact‐dependent and independent mechanisms (Figure 2) (Alvarez et al. 2018). The former includes the modulation of APC function and the induction of apoptosis in target cells, whereas the latter is characterized by the secretion of inhibitory cytokines and metabolic disruption (Zhang et al. 2021).

### 2.2 Regulation of Antigen‐Presenting Cell (APC) Maturation and Functionality

Tregs regulate APC maturation and immune function through the expression of cytotoxic T‐lymphocyte‐associated protein 4 (CTLA‐4), which competes with CD28 for binding to the costimulatory molecules CD80 and CD86 on APCs. This inhibits T cell responses by downregulating costimulatory signals (Walker 2013). It is a widely recognized key immune checkpoint and serves as a therapeutic target in the fields of autoimmunity and oncology. The interaction of CTLA‐4 with CD80 and CD86 on APCs inhibits T‐cell responses by preventing T‐cell proliferation and the production of IL‐2 (Walker 2013). CTLA‐4 also induces the expression of indoleamine 2,3‐dioxygenase (IDO) in DCs, which catalyzes tryptophan degradation, creating a metabolically hostile environment for effector T cells, leading to their starvation and apoptosis (Alvarez et al. 2018). Additionally, Tregs express lymphocyte activation gene‐3 (LAG‐3/CD223), which binds with higher affinity to major histocompatibility complex (MHC) class II molecules than the CD4 receptor on naive T cells (Wardell et al. 2021). The APC then receives inhibitory signals that reduce its activity. As a result, it exhibits lower levels of costimulatory molecules, such as CD80 and CD86, and secretes fewer inflammatory cytokines. This decreases T cell activation and weakens the immune response (Wardell et al. 2021).

### 2.3 Induction of Cytolysis or Apoptosis in Target Cells

**Figure 3:**
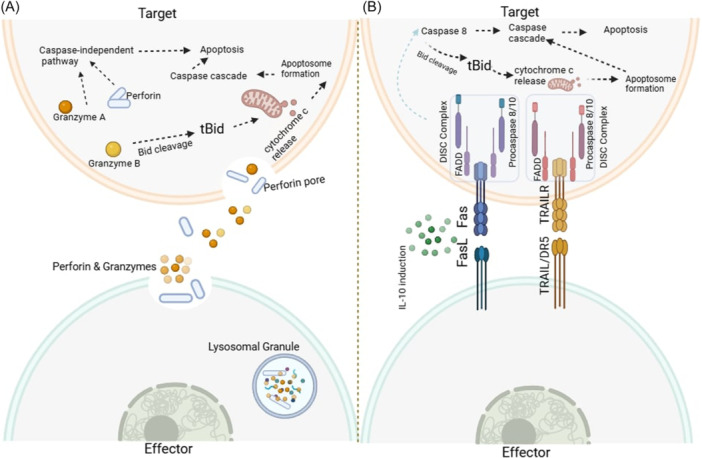
cyTregs modulate apoptosis in target cells. (A) Granzyme and perforin pathway. (B) Fas/FasL and TRAIL/TRAILR pathway.

Cytolytic Tregs (cyTregs) mediate apoptosis in target cells via perforin and granzyme pathways (Figure 3). Perforin forms pores in the target cell membrane, allowing the entry of granzymes, which induce apoptosis by disrupting mitochondrial function and activating caspases (Thiery et al. 2011).

Granzyme A (GzA) and granzyme B (GzB) induce cell death through different mechanisms. Apoptosis caused by GzA occurs independently of caspases and is characterized by a loss of cell membrane integrity, DNA damage, and mitochondrial dysfunction (Susanto et al. 2013). GzA specifically targets intracellular components, including the mitochondrial complex I component, NADH: Ubiquinone Oxidoreductase Iron‐Sulfur Protein 3 (NDUFS3). This disrupts mitochondrial metabolism, leading to an increase in the production of ROS (Lieberman 2010).

GzB triggers apoptosis via the BH3‐interacting domain death agonist (BID). BID plays a crucial role in mitochondrial permeabilization and the activation of caspases. The apoptosis pathway induced by GzB involves the clustering of mitochondria around the nucleus, mediated by BID, and the subsequent release of cytochrome c. This process leads to a caspase‐dependent cascade characterized by a decrease in mitochondrial membrane potential, nuclear condensation, and cell shrinkage (Zhang et al. 2021; Cao et al. 2007).

Tregs also utilize the tumor necrosis factor‐related apoptosis‐inducing ligand (TRAIL)/death receptor (DR) pathway to induce apoptosis. TRAIL binds to DR4 and DR5 on target cells, recruiting Fas‐associated protein with a death domain (FADD) and activating caspase‐mediated cell death (Yuan et al. 2018). The Fas/FasL pathway similarly mediates apoptosis, with IL‐10 enhancing this process by activating caspase‐8 and regulating immune homeostasis in periodontitis (Li et al. 2013).

### 2.4 Inhibition via Metabolic Disruption

Tregs suppress inflammation in periodontitis by modulating extracellular nucleotide metabolism. High ATP concentrations act as a pro‐inflammatory danger‐associated molecular pattern (DAMP), activating the NLRP3 inflammasome and promoting cytokine release (Didilescu et al. 2024). Tregs express CD39, which degrades ATP and ADP into AMP, and CD73, which converts AMP into adenosine. Adenosine then binds to A2A receptors on immune cells, increasing intracellular cyclic AMP (cAMP) and suppressing pro‐inflammatory cytokine production (Bopp et al. 2007). This metabolic regulation contributes to immune tolerance and periodontal tissue homeostasis.

### 2.5 Induction of Inhibitory Cytokines

Tregs produce key inhibitory cytokines, including IL‐35, IL‐10, and TGF‐β, which modulate immune responses in periodontal disease. IL‐35 enhances Treg proliferation while inhibiting effector T cell differentiation, particularly the pro‐inflammatory Th17 subset, thereby reducing IL‐17‐mediated inflammation (Wang and Lei 2021; Jin et al. 2017). IL‐10 downregulates MHC and co‐stimulatory molecule expression on APCs, reducing inflammatory cytokine production and suppressing APC activity (Alroqi and Chatila 2016). TGF‐β further restricts APC function and cytotoxic T cell proliferation while promoting immune tolerance.

Tregs also facilitate tissue repair by secreting amphiregulin (AREG), which binds to the epidermal growth factor receptor (EGFR) and promotes wound healing (Alvarez et al. 2018). Additionally, CTLA‐4 expressed by Tregs inhibits osteoclast differentiation and activation, preventing inflammation‐induced bone loss through the secretion of anti‐inflammatory cytokines such as IL‐4, IL‐10, and TGF‐β (Zhu et al. 2020). IL‐2, another cytokine produced by Tregs, plays a crucial role in maintaining Foxp3 expression, which is essential for Treg function. IL‐2 prevents effector T cell differentiation into inflammatory subsets and influences critical Treg marker molecules such as GITR and CTLA‐4 (Goldstein et al. 2013). Exogenous IL‐2 has been proposed to support Treg survival and enhance their immunosuppressive functions (Alvarez et al. 2018).

## 3 Th17/Treg Balance in Periodontitis

The balance between Th17 and Treg cells is crucial for maintaining periodontal health. Imbalances in these cell populations contribute to periodontitis pathogenesis (Gao et al. 2017). One key factor is the oral microbiota. A mouse model study showed that antibiotic‐induced microbiota alterations increased Th17 cell numbers and exacerbated alveolar bone resorption, suggesting that microbiota disruption promotes Th17 cell production and worsens periodontal disease (Swanson et al. 2021).

APCs, such as DCs and macrophages, recognize bacterial DNA (CpG motifs) and lipopolysaccharides (LPS) via TLRs, activating the TLR/MyD88 signaling pathway. This promotes Th17 polarization and inflammatory responses (Dutzan et al. 2018). Increased Th17 cell numbers, often with reduced Tregs, have been linked to pathogenic bacteria, including *P. gingivalis, T. forsythia, T. denticola, and Aggregatibacter actinomycetemcomitans*. However, dysbiosis, rather than a single bacterial species, likely drives Th17 induction (Dutzan et al. 2018).

Another mechanism influencing Th17/Treg balance is the “Treg/IDO axis.” IDO, expressed by DCs, regulates T‐cell responses and promotes Treg expansion (Bracho‐Sanchez et al. 2019). IDO‐mediated tryptophan degradation also activates aryl hydrocarbon receptors, enhancing Treg expansion (Van der Leek et al. 2017). Additionally, *P. gingivalis* LPS (Pg‐LPS) can upregulate IDO expression, fostering Treg expansion. However, excessive exposure shifts the balance toward Th17 dominance by promoting pro‐inflammatory cytokine secretion from DCs (Yang et al. 2017).

Cytokines regulate CD4+ T‐cell differentiation. IL‐1, IL‐6, and IL‐23 drive Th17 differentiation (Chen and O'Shea 2008), while TGF‐β, IL‐2, IL‐10, and IL‐35 enhance Treg function (Palomares et al. 2014). IL‐6 is particularly critical, converting Tregs into Th17 cells (Komatsu et al. 2014). During infection, periodontal ligament cells produce IL‐6 in response to bacterial components, triggering Treg conversion into exFoxp3Th17 cells. A study found that anti‐IL‐6 receptor antibodies significantly inhibited this process (Komatsu et al. 2014).

### 3.1 RANKL/RANK/OPG and Periodontal Bone Resorption

Bone remodeling is regulated by the coordinated actions of osteoclasts and osteoblasts (Belibasakis and Bostanci 2012). In periodontitis, this balance is disrupted, leading to excessive osteoclast activity and net alveolar bone loss (Belibasakis and Bostanci 2012; Crotti et al. 2003). Receptor activator of nuclear factor‐κB ligand (RANKL), encoded by TNFSF11, promotes osteoclast differentiation by binding RANK on osteoclast precursors in the presence of macrophage colony‐stimulating factor (M‐CSF) (Belibasakis and Bostanci 2012). RANKL is produced not only by osteoblasts, but also by periodontal ligament fibroblasts, gingival fibroblasts, and activated T and B cells within inflamed periodontal tissues (Belibasakis and Bostanci 2012; Crotti et al. 2003). Pro‐inflammatory cytokines such as IL‐1β, TNF‐α, and IL‐17 further upregulate RANKL expression and downregulate OPG, a soluble decoy receptor that binds RANKL and prevents its interaction with RANK (Adamopoulos et al. 2010; Song et al. 2019). Consequently, an increased RANKL/OPG ratio is consistently observed in periodontal lesions and correlates with clinical measures of disease severity, linking aberrant immune activation directly to osteoclastogenesis and alveolar bone resorption (Belibasakis and Bostanci 2012; Crotti et al. 2003).

### 3.2 Th17/Treg Imbalance and Bone Resorption

Th17 cells contribute to bone resorption through IL‐17, which upregulates RANK expression on osteoclast precursors, enhancing their sensitivity to RANKL (Adamopoulos et al. 2010; Song et al. 2019). However, findings on IL‐17's effect on osteoclastogenesis are inconsistent, with some studies reporting suppression of osteoclast differentiation (Wijekoon et al. 2017). Experimental models using IL‐17 receptor knockout mice suggest that IL‐17 deficiency worsens alveolar bone loss due to reduced neutrophil accumulation (Gaffen and Hajishengallis 2008). These discrepancies highlight the complexity of the role of IL‐17.

In contrast, Tregs exert anti‐osteoclastogenic effects via cytokines such as TGF‐β, IL‐10, and IL‐35 (Garlet et al. 2010). IL‐10, in particular, suppresses the IL‐17‐mediated inflammatory response and lowers the RANKL/OPG ratio. IL‐10‐deficient mice with periodontitis show increased IL‐17 expression and more severe bone loss (Sun et al. 2020). IL‐35 also counteracts Th17/Treg imbalance, providing both osteoprotective and immunoregulatory effects. Although IL‐35 has shown regulatory and osteoprotective effects in experimental periodontitis models by restoring the Th17/Treg balance, no human clinical trials have yet evaluated IL‐35–based therapies, and its potential for inducing systemic immunosuppression requires careful consideration before clinical translation (Schmidlin et al. 2021; Jin et al. 2017).

Among T‐cell subsets, exFoxp3Th17 cells, derived from Tregs in the presence of IL‐6, exhibit the highest RANKL expression (Tsukasaki et al. 2018; Komatsu et al. 2014). These cells contribute significantly to periodontal bone loss by inducing RANKL expression in osteoblasts and periodontal ligament cells (Lin et al. 2015). This pathogenic Treg‐to‐Th17 conversion is a key driver of bone resorption in periodontitis (Tsukasaki et al. 2018).

## 4 Therapeutic Application of Tregs

### 4.1 IL‐2 as a Major Growth Factor for Tregs

Interleukin‐2 (IL‐2) is a crucial cytokine that plays an indispensable role in the maintenance and expansion of regulatory T cells (Tregs). Tregs express high levels of the IL‐2 receptor alpha chain (CD25), which allows them to effectively utilize low concentrations of IL‐2, unlike conventional effector T cells. IL‐2 binding to its receptor on Tregs induces the transcription of FOXP3, the master regulator responsible for Treg differentiation and function (Sakaguchi et al. 2008). This mechanism not only supports the survival and functional stability of Tregs, but also enhances their suppressive abilities. In the context of periodontitis, the administration of low‐dose IL‐2 has been proposed as a potential therapeutic strategy to boost Treg populations and restore immune homeostasis, thereby controlling excessive inflammation and bone resorption (Whiteside 2015). Increasing Treg numbers in periodontitis may counterbalance the inflammatory Th17 responses, which are typically elevated in periodontal disease.

However, the therapeutic use of IL‐2 is not without challenges. IL‐2 also promotes the growth of effector T cells, including pro‐inflammatory subsets such as Th1 and Th17 cells, which could potentially exacerbate inflammation in periodontitis. Therefore, precise modulation of IL‐2 doses is necessary to selectively expand Tregs without activating pathogenic effector T cells. Recent advances in IL‐2‐based therapies involve engineered IL‐2 variants that selectively target Tregs, providing a promising avenue for developing Treg‐based therapies in chronic inflammatory conditions like periodontitis (Ye et al. 2018).

Beyond cytokine‐based modulation, additional Treg‐centered strategies have been investigated in chronic inflammatory diseases, including adoptive transfer of ex vivo–expanded Tregs has shown promise in restoring immune tolerance in chronic inflammatory conditions (Amato et al. 2022; Zhang et al. 2022). In addition, in vivo expansion of Tregs using low‐dose IL‐2 selectively enhances FOXP3⁺ regulatory populations and represents an emerging immunomodulatory approach (Alroqi and Chatila 2016). These approaches enhance Treg suppressive capacity and may represent future therapeutic directions for periodontitis.

### 4.2 FOXP3 and Co‐Inhibitory Receptors

FOXP3+ Tregs suppress immune responses through multiple mechanisms, one of which involves the expression of co‐inhibitory receptors such as cytotoxic T‐lymphocyte‐associated protein 4 (CTLA‐4) and glucocorticoid‐induced tumor necrosis factor receptor (GITR). These molecules are critical for maintaining immune tolerance by inhibiting the activation of effector T cells and DCs. CTLA‐4, in particular, binds to the CD80/CD86 receptors on APCs, thereby preventing the co‐stimulation necessary for T cell activation. The therapeutic targeting of CTLA‐4 has been explored in various inflammatory conditions, including periodontitis, with the goal of enhancing Treg function to suppress excessive inflammation (Oparaugo et al. 2023). Furthermore, GITR signaling enhances the proliferation of Tregs while simultaneously inhibiting the function of effector T cells, making it another potential target for therapeutic interventions in periodontitis.

In addition to CTLA‐4 and GITR, Tregs also produce anti‐inflammatory cytokines such as IL‐10 and transforming growth factor‐beta (TGF‐β), which directly suppress pro‐inflammatory responses and promote tissue repair. The modulation of these pathways in periodontitis could help restore tissue homeostasis by limiting osteoclast activity and bone destruction while preserving the protective roles of Tregs. Hence, therapies that boost FOXP3 expression or enhance the activity of co‐inhibitory receptors could provide effective means to manage the immune dysregulation seen in periodontitis (Fontenot et al. 2003).

### 4.3 All‐Trans Retinoic Acid (ATRA) and IL‐33 in Reinforcing Treg Functions

All‐trans retinoic acid (ATRA), a metabolite of vitamin A, has been demonstrated to enhance the differentiation and suppressive function of Tregs. ATRA exerts its effects by promoting FOXP3 expression and inhibiting the differentiation of pro‐inflammatory Th17 cells (Mucida et al. 2009). Given that the Th17/Treg imbalance plays a significant role in the pathogenesis of periodontitis, ATRA represents a promising therapeutic agent. Studies have shown that ATRA, when used in animal models of periodontal disease, reduces alveolar bone loss by enhancing the function of Tregs and dampening inflammatory cytokine production (Wang et al. 2014). Moreover, ATRA's ability to skew the Treg/Th17 balance towards an anti‐inflammatory phenotype suggests its potential application in controlling periodontal inflammation and preventing tissue destruction.

IL‐33, another immunomodulatory cytokine, has emerged as a potent enhancer of Treg function. IL‐33 binds to the ST2 receptor on Tregs and promotes their suppressive activities, leading to increased production of IL‐10 and TGF‐β (Schiering et al. 2014). In the context of periodontitis, IL‐33 has been shown to mitigate alveolar bone loss by modulating the local immune environment and promoting tissue repair. IL‐33 also recruits Tregs to sites of inflammation, enhancing their immunosuppressive capabilities. The combined use of IL‐33 and ATRA could potentially synergize to enhance Treg‐mediated suppression in periodontal disease, offering a novel therapeutic approach to controlling chronic inflammation and tissue degradation.

### 4.4 CCL22 and Treg Recruitment

Chemokine ligand 22 (CCL22) is another molecule that plays a pivotal role in the recruitment of Tregs to sites of inflammation. Tregs express the chemokine receptor CCR4, which binds to CCL22, facilitating their migration to inflamed tissues. One study has demonstrated that enhancing CCL22‐mediated recruitment of Tregs to periodontal lesions could help in controlling local inflammation and preventing periodontal tissue destruction (Glowacki et al. 2013). Thus, increased Treg infiltration in periodontal tissues is associated with reduced inflammatory responses and lower levels of bone resorption.

However, the therapeutic targeting of chemokines like CCL22 must be approached with caution, as excessive Treg recruitment could potentially repress protective immune responses necessary for controlling bacterial infections by suppressing B‐lymphocytes (Lim et al. 2005). Therefore, balancing Treg recruitment while maintaining effective immune surveillance is critical in designing chemokine‐based therapies for periodontitis (Table 1).

**Table 1:** Features of different Tregs subsets in periodontitis.

Feature	nTregs	pTregs	Tr1	CD8 ++Tregs	IL‐17 producing Tregs
Origin	Thymus‐in response to self‐antigens	Periphery‐in response to foreign antigens	Periphery‐under chronic antigen exposure	Thymus or periphery‐under chronic antigen exposure or immune stress	Periphery‐under inflammatory or infectious settings
Signature transcription factor	FoxP3+ expression	FoxP3+ expression	No FoxP3 expression	Variable FoxP3 expression	FoxP3 and RoRγt dual expression
Key cytokines	IL‐10, TGF‐β	IL‐10, TGF‐β	IL‐10	IL‐10, TGF‐β, Granzyme B	IL‐17, IL‐10, TGF‐β
Regulatory function in periodontitis	Strong regulator of immune tolerance and prevention of autoimmunity. nTregs secrete anti‐inflammatory cytokines, such as IL‐10, TGF‐β and IL‐35, which mitigate the inflammatory response and promote tissue repair and IL‐10 inhibits the activation of antigen‐presenting cells (APCs) and reduces the release of pro‐inflammatory mediators. Also, suppress the RANKL pathway and promote the OPG expression	Regulator of immune tolerance and mucosal immunity. Mitigate the activity of pro‐inflammatory T cells and reduce the production of cytokines like IL‐17 and IFN‐γ that exacerbate periodontal tissue destruction. Also, regulate the RANKL‐OPG axis.	Control of chronic inflammationTr1 cells secrete IL‐10, a potent anti‐inflammatory cytokine that suppresses the activity of pro‐inflammatory immune cells such as Th1, Th17 cells, macrophages, and dendritic cells.	Regulation of cytotoxic responses. Prevent excessive tissue damage caused by CD8⁺ cytotoxic T lymphocytes. CD8⁺ Tregs inhibit the proliferation and activity of effector T cells via both contact‐dependent mechanisms (e.g., CTLA‐4, PD‐1) and via IL‐10 and TGF‐β.	Plasticity under inflammatory conditions. While IL‐17 promote osteo‐clastogenesis and bone resorption, IL‐10 and TGF‐β counteract these effects. This dual action helps maintain a balance between inflammation needed to manage infection and the prevention of excessive bone loss in periodontitis.

## 5 Conclusion

Regulatory T cells (Tregs) play a critical role in maintaining immune homeostasis and preventing tissue destruction in chronic inflammatory diseases such as periodontitis. Their therapeutic potential lies in their ability to suppress pro‐inflammatory responses, promote tissue repair, and inhibit osteoclastogenesis. IL‐2, ATRA, IL‐33, and chemokine pathways like CCL22 offer promising strategies for enhancing Treg function and restoring immune balance in periodontitis. However, challenges remain in selectively expanding Tregs without activating pro‐inflammatory effector cells. Future research should focus on refining these therapeutic strategies to optimize their efficacy and safety in clinical settings. By leveraging the immunosuppressive and tissue‐protective properties of Tregs, it may be possible to develop more effective treatments for periodontitis, ultimately improving patient outcomes and preserving oral health.

## Author Contributions

Revan Birke Koca Ünsal, Akira Hasuike, Farah Asa'ad, Bruno Špiljak, Tamer Badawy, Jelena Roganović, Yinli Liu, and Carel Brigi contributed to conception, data acquisition, and interpretation. Carel Brigi and Jelena Roganović contributed to figures and table. Revan Birke Koca Ünsal contributed to overall manuscript organization and final draft preparation. Akhilanand Chaurasia contributed to conception, design, critically revised the manuscript and submission. All authors gave their final approval and agree to be accountable for all aspects of the work.

## Funding

The authors received no specific funding for this work.

## Ethics Statement

The authors have nothing to report.

## Consent

The authors have nothing to report.

## Conflicts of Interest

The authors declare no conflicts of interest.

## Data Availability

Data sharing not applicable to this article as no datasets were generated or analyzed in this narrative review.
